# Neoadjuvante Radiochemotherapie verbessert bei resektablen und grenzwertig resezierbaren Pankreaskarzinomen die onkologischen Ergebnisse, bei resezierbaren aber nicht das Gesamtüberleben: Ergebnisse der niederländischen PREOPANC-Studie

**DOI:** 10.1007/s00066-020-01717-6

**Published:** 2020-11-30

**Authors:** Robert Michael Hermann, Hans Christiansen

**Affiliations:** 1 Zentrum für Strahlentherapie, Mozartstr. 30, 26655 Westerstede, Deutschland; 2 Klinik für Strahlentherapie und Spezielle Onkologie, Medizinische Hochschule Hannover, Carl-Neuberg-Str. 1, 30625 Hannover, Deutschland

## Zielsetzung

Mit einer randomisierten Studie sollte der Stellenwert einer neoadjuvanten Radiochemotherapie (RCTX) bei resektablen und fraglich resektablen Pankreaskarzinomen im Vergleich zu einer primären Operation gefolgt von einer adjuvanten Chemotherapie (CTX) geprüft werden. Ziel der Vorbehandlung war ein Downstaging, das zu einer Verbesserung der operativen Ergebnisse und letztlich auch der Prognose führen kann. Ein weiteres Argument für die neoadjuvante Therapie war die meist bessere Compliance im Vergleich mit der adjuvanten Therapie nach großen tumorchirurgischen Eingriffen. Nachteilig hingegen könnten sich eine höhere perioperative Letalität und eine nicht ausreichend sichere kurative Resektion auswirken mit der Folge eines Progresses unter der adjuvanten Therapie.

## Studiendesign

Prospektiv wurden zwischen 2013 und 2017 in den Niederlanden 246 Patienten in der PREOPANC-Studie behandelt. Einschlusskriterien waren hämatogen nichtmetastasierte Pankreaskarzinome. Tumoren ohne Involvierung der Arterien und nur mit <90° Ummauerung der Venen wurden als „resektabel“ eingestuft, Tumoren mit Involvierung der Arterien <90° und/oder der Venen zwischen 90 und 270° (ohne Okklusion) als grenzwertig resektabel. Ausgeschlossen waren cT1-Tumoren.

Die Patienten wurden randomisiert zwischen einer präoperativen RCTX („Neoadjuvanz“) und einer primären Operation gefolgt von einer adjuvanten CTX („Kontrolle“). Im neoadjuvanten Arm wurde zum Ausschluss einer okkulten Metastasierung und zur Beurteilung der Resektabilität vor Einleitung der Therapie eine Staging-Laparoskopie durchgeführt. Die RCTX bestand aus 3 Zyklen Gemcitabin (1000 mg/m^2^ d1, 8, 15), der zweite Zyklus begleitet von einer Radiotherapie (RTX) mit 15 × 2,4 Gy auf Primärtumor und verdächtige Lymphknoten. Nach 4 Wochen erfolgte ein Restaging mittels CT und nach 14–18 Wochen eine explorative Laparotomie, möglichst gefolgt von der Tumorresektion; postoperativ wurden noch 4 Zyklen Gemcitabin ergänzt. Im Kontrollarm wurden unmittelbar nach der Operation adjuvant 6 Zyklen Gemcitabin gegeben. Bei Pankreaskopfkarzinomen wurde eine pyloruserhaltende Whipple-Operation, bei Karzinomen des Pankreaskörpers und -schwanzes eine subtotale Organresektion mit Splenektomie durchgeführt.

Primärer Endpunkt war das Gesamtüberleben (OS), sekundäre Endpunkte das krankheitsfreie Überleben (DFS), die lokoregionale Kontrolle, die Fernmetastasenfreiheit, die Resektionsraten (R0–R2) und schließlich die Toxizität. Bei der statistischen Aufplanung der Studie wurde davon ausgegangen, durch die Neoadjuvanz das mediane Überleben von 11 auf 17 m (Monate) verlängern zu können.

## Ergebnisse

56 % der Patienten waren männlich und im Median 66 Jahre alt und in gutem Performance-Status. Bei 87 % waren die Karzinome im Pankreaskopf lokalisiert. Etwas mehr als die Hälfte der Karzinome wurde als resektabel, die anderen als grenzwertig resektabel eingestuft. 29 % hatten klinisch befallene regionale Lymphknoten. Im neoadjuvanten Therapiearm begannen nur 91 von 119 Patienten (76 %) mit der RCTX, weil die anderen u. a. peritoneale Metastasen bei der Staging-Laparoskopie hatten. 81 (89 %) erhielten die RCTX vollständig, vorzeitig wurde z. B. wegen Toxizität und dreimal wegen klinischen Progresses abgebrochen, zwei Todesfälle traten auf. Im CT beim Restaging fand sich bei 10 Patienten ein Progress. Letztlich wurden 72 (61 % des Gesamtkollektivs) reseziert. Die Resektionsraten unterschieden sich nach Neoadjuvanz und in der Kontrollgruppe nicht signifikant. In der Kontrollgruppe wurden 92 von 127 Patienten (72 %) reseziert, u.a. 14 Patienten nicht wegen okkulter Metastasen in der Staging-Laparoskopie. Die R0-Resektions-Rate war jedoch nach Neoadjuvanz signifikant höher als in der Kontrollgruppe (71 % vs. 40 %), dort war auch der Lymphknotenbefall signifikant geringer (33 % vs. 78 %), ebenso das perineurale Wachstum 39 % vs. 73 % und die venöse Infiltration 19 % vs. 36 %. Die adjuvante Chemotherapie begannen 55 Patienten (46 %) in der Neoadjuvanzgruppe, verglichen mit 51 % bei der Kontrollgruppe.

Nach 27 m (Monaten) Follow-up betrug das mediane OS in der Neoadjuvanz 16 m, verglichen mit 14,3 m in der Kontrollgruppe (n.s.). Dabei lag das OS bei den Kontrollen oberhalb der Annahmen bei der statistischen Studienplanung. Allerdings verbesserte die Neoadjuvanz das DFS und die lokale Kontrolle signifikant bei vergleichbarer Fernmetastasenfreiheit. Der deutlichste Effekt – auch auf das OS bezogen – zeigte sich in der Subgruppe der grenzwertig resektablen Karzinome: Hier wurde durch die Neoadjuvanz das mediane OS von 13 auf 17,6 m signifikant verlängert und zudem die lokale Kontrolle von 11 auf 27 m mehr als verdoppelt. Die Fernmetastasenfreiheit betrug 21,5 m im Vergleich zu 12,2 m in der Kontrollgruppe.

In beiden Armen war das OS nach R0-Resektionen signifikant besser als nach R1/2.

Hinsichtlich der Toxizität wurden in beiden Armen vergleichbar oft „serious adverse events“ (SAE) gemeldet (52 % vs. 41 %). Die perioperative Letalität wurde durch die Neoadjuvanz nicht gesteigert. Allerdings erhöhte sich die postoperative Komplikationsrate von 50 auf 68 % (*p* = 0,26).

## Schlussfolgerungen der Autoren

Das primäre Studienziel, nämlich eine signifikante Verbesserung des OS durch die Neoadjuvanz im Gesamtkollektiv, wurde zwar verfehlt, doch wurden – bei insgesamt auch besserer Compliance – alle sekundären Endpunkte verbessert, sodass das neoadjuvante Therapieschema im Vergleich zur primären Operation gefolgt von einer adjuvanten CTX überlegen erscheint.

## Kommentar

Die hier vorgestellte niederländische Studie PREOPANC imponiert als erste große prospektiv randomisierte Studie, die den Stellenwert einer neoadjuvanten RCTX bei resektablen bzw. grenzwertig resektablen Pankreaskarzinomen herausarbeitete und publizierte.

Dabei zeigte sich – trotz des verfehlten primären Endpunkts der Verbesserung des OS im gesamten Kollektiv – eine beeindruckende Wirkung der neoadjuvanten Therapie. Das erhoffte Downstaging war so ausgeprägt, dass die prognostisch wichtige Rate an R0-Resektionen fast verdoppelt und die lymphogene Metastasierung nahezu halbiert wurde. Als „proof of principle“ können dabei die Therapieeffekte bei den Patienten mit grenzwertig resektablen Karzinomen gelten: Durch die Vorbehandlung wurden diese Tumoren deutlich sicherer resektabel, was sich über die massiv verbesserte lokale Kontrolle bis in ein signifikant verlängertes OS übersetzte. Neben der verbesserten lokalen Situation bei der Operation mag dabei auch entscheidend sein, dass drei Viertel der Patienten in der Neoadjuvanz zumindest mit der Systemtherapie begannen und fast 90 % dieser Patienten die Therapie auch vollständig erhielten. Nach primärer Operation konnte nur bei der Hälfte der Patienten postoperativ trotz des Settings einer großen Studie in tertiären Hochvolumenzentren die adjuvante CTX eingeleitet werden. In Kombination mit Gemcitabin war die Toxizität der RCTX dabei gut zu handhaben: Zwar war die postoperative Morbidität etwas erhöht, nicht aber die Letalität.

Eine kleinere koreanische Studie war zwei Jahre zuvor zu einem ähnlichen Ergebnis gekommen und unterstützt somit den Befund der PREOPANC-Studie, auch wenn die statistische Aussagekraft bei nur 50 randomisierten Patienten deutlich geringer ist wegen der vorzeitigen Schließung der Studie [1]. Dabei wurde ebenfalls eine Gemcitabine-basierte RCTX appliziert, die RTX erfolgte normofraktioniert bis 54 Gy sowohl im neoadjuvanten als auch im adjuvanten Arm. Das mediane OS wurde durch die Neoadjuvanz signifikant von 12 auf 21 m verbessert, ebenso die R0-Resektionen von 26 auf 52 % erhöht. Eine große Metaanalyse mit fast 3500 Patienten in retrospektiven und prospektiven Serien mit überwiegend resektablen Karzinomen zeigte ebenfalls eine Verlängerung des OS von 15 auf 19 m durch eine neoadjuvante (R)CTX, verglichen mit einem primär operativen Vorgehen ohne Neoadjuvanz [2].

Aus radioonkologischer Sicht ist das pragmatische Vorgehen bei der Bestrahlungsplanung in PREOPANC interessant. Vollständig wurde auf eine Erfassung elektiver Lymphabflüsse verzichtet. Als GTV wurde der Primärtumor einschließlich eventuell vergrößerter Lymphknoten gewählt, möglichst auf einem 4D-CT mit 10 Atemphasen abgegrenzt. Zum CTV wurde das GTV um 5 mm erweitert, das ITV stellte die Überlagerung der CTV in allen Atemphasen dar. Dieses wurde schließlich zum PTV um 1 cm erweitert. Aufgrund der Dosisvorgaben werden damit die „constraints“ der meisten Risikoorgane relativ problemlos eingehalten.

Trotz der Unterstützung durch die angeführten Vorgängerstudien lassen sich die Vorgaben und Ergebnisse der PREOPANC-Studie nicht unmittelbar in unsere klinische Praxis überführen. Die Systemtherapien sind mittlerweile deutlich effektiver geworden als die Monotherapie mit Gemcitabin. Zudem wurde im neoadjuvanten Setting nur ein Teil der Systemtherapie appliziert. Um die Vorteile des präoperativen Settings maximal ausnutzen zu können, wäre ein Protokoll im Sinne einer „vollständigen neoadjuvanten Therapie“ notwendig gewesen. Die derzeit besten Studienergebnisse sind bei resektablen Tumoren für die primäre Operation gefolgt von modifiziertem FOLFIRINOX publiziert worden, allerdings in einem hochselektionierten Patientengut (nach guter Genesung von der Tumorresektion), weswegen die Daten mit der hier vorgestellten Studie nicht verglichen werden können [3]. Der Logik der Überlegenheit neoadjuvanter Therapiestrategien folgend, rekrutieren derzeit mehrere Studien, die überwiegend in resektablen Stadien FOLFIRINOX neoadjuvant gegen adjuvant testen: NorPACT‑1 [ClinicalTrials.gov NCT02919787], PANACHE01-PRODIGE48 [NCT02959879], PREOPANC‑2. Auch andere Kombinationstherapien werden aktuell klinisch auf die optimale Sequenz hin untersucht (z. B. Gemcitabin + nab-Paclitaxel durch die AIO-Studie NEONAX [NCT02047513]).

Mit Blick auf die in der hier kommentierten PREOPANC-Studie erfolgte ausgeprägte Tumorregression durch die neoadjuvante RTX, allein schon in Kombination mit Gemcitabin, erscheint als nächster Schritt der Therapieoptimierung die Integration der RTX in moderne neoadjuvante Polychemotherapieprotokolle sinnvoll. Und das insbesondere bei grenzwertig resektablen bzw. primär nichtresektablen, lokal fortgeschrittenen Tumoren, um sie in eine kurativ operable Situation zu überführen. Solche Therapieschemata sind bereits auf ihre Durchführbarkeit getestet, z. B. bei grenzwertig resektablen Tumoren mit einer 65 %igen R0-Resektions-Rate [4]. Bisher kann ihr Stellenwert aber solange sicher eingeordnet werden, bis Phase-III-Daten publiziert sind. Bei primär nichtresektablen Karzinomen zeigten die ersten Zwischenauswertungen der CONKO-007-Studie bei 180 Patienten, dass nach einer Induktionstherapie mit FOLFIRINOX oder Gemcitabin immerhin noch 70 % anschließend zwischen weiterer CTX oder RCTX randomisiert werden konnten [5]. Insgesamt wurden 20 % des Gesamtkollektivs schließlich einer Resektion zugeführt, und immerhin konnten 14 % doch noch R0-reseziert werden, mit entsprechend verbesserter Prognose im Vergleich mit nichtoperierten Patienten.

## Fazit

1. Durch die neoadjuvante Radiochemotherapie (RCTX) mit Gemcitabin wurde im Vergleich zur primären Operation gefolgt von einer adjuvanten Chemotherapie (CTX) zwar noch nicht das „overall survival“ (OS) des Gesamtkollektivs verbessert, aber immerhin ein ausgeprägtes Downstaging erreicht, welches zu einer deutlichen Erhöhung der R0-Resektions-Rate, der lokalen Kontrolle und des DFS führte.
2. Patienten mit grenzwertig R0-resektablen Pankreaskarzinomen profitierten besonders von der neoadjuvanten RCTX: In dieser Subgruppe kann sogar das OS signifikant verlängert werden.
3. Die neoadjuvante Therapie ist in erfahrenen Händen gut steuerbar und verträglich. Zwar kann sie zu erhöhter postoperativer Morbidität führen, aber wohl kaum zu einer gesteigerten Letalität.
4. Der Stellenwert der RTX im Setting moderner Polychemotherapien (FOLFIRINOX) kann noch nicht sicher abgeschätzt werden. Zudem wird wahrscheinlich eine differenzierte Betrachtung der resektablen, grenzwertig resektablen und nichtresektablen, aber noch lokoregionär begrenzten Tumoren notwendig sein.

*Robert Michael Hermann, Westerstede, und Hans Christiansen, Hannover*
